# Integrating neurobiological markers to prospectively predict adolescent non-suicidal self-injury and suicide attempts: a machine learning approach

**DOI:** 10.1186/s13034-026-01122-4

**Published:** 2026-06-22

**Authors:** Corinna Reichl, Erik Fink, Luisa von den Driesch, Stefan Lerch, Julian Koenig, Thomas Berger, Michael Kaess

**Affiliations:** 1 University Hospital of Child and Adolescent Psychiatry and Psychotherapy, University of Bern, Bolligenstrasse 111, Bern 60, 3000 Switzerland; 2 Department of Child and Adolescent Psychiatry, Psychosomatics and Psychotherapy, Faculty of Medicine, University Hospital Cologne, University of Cologne, Cologne, Germany; 3 Department of Clinical Psychology and Psychotherapy, University of Bern, Bern, Switzerland; 4 Department of Child and Adolescent Psychiatry, Centre for Psychosocial Medicine, University of Heidelberg, Heidelberg, Germany; 5 Graduate School of Health Sciences, University of Bern, Bern, Switzerland

**Keywords:** Suicidal behavior, Non-suicidal self-injury, Adolescents, Neuroendocrine markers, Machine learning, Longitudinal study

## Abstract

**Background:**

Suicide attempts (SA) and non-suicidal self-injury (NSSI) are closely related phenomena, both common in adolescent clinical samples. This study examined whether neurobiological markers could enhance the prediction of SA and NSSI over a 2-year period, beyond established clinical predictors and age, in a high-risk sample of female adolescents engaging in NSSI.

**Methods:**

A total of *n* = 63 (age: *M* = 15.1 years, *SD* = 1.45) female adolescents with NSSI were recruited from our outpatient clinic for risk-taking and self-harming behaviour (AtR!Sk) at the Clinic for Child and Adolescent Psychiatry, University Hospital of Heidelberg, Germany. Machine learning models (linear and logistic regression; elastic net regression; support vector machines with repeated cross-validation) were applied. We tested whether the inclusion of biomarkers (thyroid-stimulating hormone [TSH]; free triiodothyronine [fT3]; adrenocorticotropic hormone [ACTH]; dehydroepiandrosterone sulfate [DHEA-S]; resting heart rate variability [rHRV]; pain threshold) improved prediction of SA and NSSI remission – defined as the absence of any NSSI episodes – over a two years period, beyond clinical variables (past SA; symptoms of borderline personality disorder [BPD]) and age.

**Results:**

Models predicting SA that included biomarkers, clinical variables, and age, showed moderate predictive accuracy (AUC: 0.76–0.82), however not higher than when only including clinical variables and age. The strongest predictors were past SA (OR = 7.28), followed by fT3 (OR = 0.16) and TSH (OR = 0.35). The SA prediction models showed high specificity but low sensitivity, indicating strong performance in identifying negative cases but poor detection of positive cases. Models predicting NSSI, including the same variables, did not consistently outperform chance levels and showed low specificity and sensitivity.

**Conclusions:**

These findings support the use of machine learning models to predict SA in high-risk adolescents but show that prediction is driven mainly by established clinical features (past SA; BPD) and age, with no added benefit from neurobiological markers. Moreover, all models were more effective at identifying negative than positive cases. Given the small sample size and class imbalance, findings should be interpreted cautiously and require replication in larger and independent samples.

**Supplementary Information:**

The online version contains supplementary material available at https://doi.org/10.1186/s13034-026-01122-4.

## Introduction

Suicide is the third most common cause of death among adolescents and young adults worldwide [1]. In Switzerland, an estimated 127 suicide attempts occur for every completed suicide among 16- to 20-year olds [2]. Risk factors for the development of suicide attempts (SA) include demographic, environmental, biological, and psychiatric variables. Younger age [3], female sex [4], childhood adversity [5], psychiatric comorbidity [6] and prior suicide attempts [7] are particularly associated with increased risk. Moreover, non-suicidal self-injury (NSSI) has been identified as a risk marker for SA. Unlike many psychiatric symptoms, NSSI is relatively observable and accessible in the clinical setting. Meta-analyses of longitudinal, population-based studies confirm a significant association between NSSI and future SA [8]. Additionally findings by Koenig et al. [9] show that NSSI prospectively predicts suicidal plans and attempts among adolescents.

Despite decades of research, the prediction of SA remains limited. Franklin et al. [10] summarized 50 years of findings and concluded that suicide attempt predictions are only marginally better than chance, with even lower accuracy in clinical samples. Chan et al. [11] reported that while prior self-harm predicts future suicide, validated risk scales do not. This presents a challenge for clinicians, as common risk factors are widespread in clinical populations and lack discriminatory power. Accordingly, Franklin et al. [10] suggested that machine-learning (ML) approaches could improve risk prediction.

ML offers advantages over traditional methods by integrating numerous predictors and modeling complex, non-linear interactions. Meta-analyses indicate ML outperforms conventional models in predicting SA [12–16]. However, Kusuma et al. [14] emphasize that current ML research largely depends on electronic health records and survey data, underscoring the need to incorporate more diverse data sources. Additionally, researchers emphasize ML’s potential to advance the identification of biomarkers for the prediction of SA [e.g., 17]. In a large population study, Zhang et al. [16] integrated physiological and behavioral variables, identifying mental health and lifestyle factors as the strongest predictors of SA. Additionally, several biomarkers – including immunological markers – were elevated in adults with SA compared to controls. Regarding the application of ML to predict suicidality in adolescence, the existing body of research remains limited. Evidence from both a meta-analysis [18] and a longitudinal study [19] suggests that such models achieve moderate predictive performance in identifying suicide attempts among adolescents. However, to our knowledge, no studies to date have incorporated a set of neurobiological variables into ML models aimed at longitudinally predicting suicide attempts within adolescent populations.

Research on using ML to predict NSSI is still emerging. Depression has been identified as one of the most important predictors of NSSI among adolescents in population-based samples [20] and in samples of adolescents with major depressive disorder [21]. In a cross-sectional study, Mürner-Lavanchy et al. [22] found a set of biomarkers (mainly low oxytocin, low pain sensitivity and high leukocytes) to have moderate predictive performance; though more complex ML models did not outperform logistic regression. Moreover, Fink et al. [23] identified specific biomarkers to cross-sectionally differentiate adolescents with NSSI and a history of SA from those with NSSI alone. To our knowledge, no study has yet applied ML to longitudinally examine the remission of NSSI, despite its established relevance as an antecedent of SA. Remission refers to the complete cessation of NSSI within a specified time frame [24], a minimum duration of one year appears appropriate, as the time frame is consistent with the DSM 5 definition of NSSI disorder [25]. Even though NSSI constitutes a complex dimensional behavior with dynamic trajectories [26], the use of NSSI remission as an outcome is supposed to capture a clinically meaningful endpoint with decision-making relevance.

This study examined whether including biomarkers (thyroid-stimulating hormone, TSH; free triiodothyronine, fT3; adrenocorticotrophic hormone, ACTH; dehydroepiandrosterone sulfate, DHEA-S; resting heart rate variability, rHRV; pain threshold) improves ML prediction of SA and NSSI remission over two years beyond common predictors. Biomarkers were selected based on their content relevance and temporal stability. Since adolescents typically engage in self-harm during periods of heightened stress [27, 28], the hypothalamic-pituitary-adrenal (HPA) axis and the autonomic nervous system (ANS) – two key stress response systems – are considered crucial in self-harm development. Both systems facilitate adaptation to dynamic environmental demands, and alterations in their basal functioning or stress reactivity may contribute to the course of NSSI and SA. Regarding HPA axis functioning, alterations have been linked to NSSI [27] and SA [28]. While most studies focus on cortisol, we included ACTH in our analyses due to its greater temporal stability in our cohort. However, studies associating ACTH to self-harm are limited, and initial findings do not support a link between ACTH levels and SA [29]. Evidence for the ANS indicates reduced vagally-mediated HRV, reflecting impaired regulatory capacity in individuals with self-harm before and after stress [30]. DHEA-S, like cortisol stimulated by ACTH, acts as both a sex and stress hormone [31]. Though its role in self-harm is unclear, initial studies report decreased DHEA-S in adults who attempted suicide compared to controls [32, 33]. The hypothalamic-pituitary-thyroid (HPT) axis, responsive to stress and linked to mental illness [34], shows alterations in adolescents with NSSI, including blunted fT3 to free thyroxine (fT4) ratios compared to healthy controls [35]. While decreased ft3 relates to SA [36], meta-analyses report mixed findings regarding TSH alterations in suicide attempters [29, 36]. Finally, the pain analgesia hypothesis [37] suggests adolescents engaging in NSSI show lower pain sensitivity, potentially increasing suicide capability [38]. The Interpersonal Theory of Suicide [39] proposes that suicidal behavior emerges from the interaction of risk factors, including an acquired capability for suicide. A central component of this capability is reduced sensitivity to physical pain, which develops through repeated exposure to painful experiences. Over time, experiences such as NSSI may habituate individuals to pain, thereby lowering pain sensitivity and diminishing the natural aversion to self-injury [40]. Accordingly, this reduced pain sensitivity helps explain the transition from suicidal ideation to suicide attempts by increasing tolerance for self-inflicted harm. Koenig et al. [41] meta-analytically confirmed a lower pain sensitivity, higher pain threshold, and tolerance in adolescents with self-harm versus healthy controls, whereas a recent meta-analysis in adults with SA found that pain sensitivity is more closely related to general psychopathology than to suicidality [42].

To sum up, the present study aimed to apply ML models to determine whether biomarkers from the neuroendocrine (ACTH; TSH, fT3; DHEA-S), and the autonomic nervous system (rHRV), as well as pain (pain threshold) predict the two-year course of SA (yes/no) and NSSI (remission vs. no remission) beyond established clinical predictors (past self-harm, SA or NSSI; BPD) and age in a high-risk sample of female adolescents with repetitive NSSI. The primary aim was to assess whether these neurobiological markers provide predictive value for future SA, including incremental value over and above clinical features. The secondary aim was to examine whether the same set of predictors can forecast NSSI remission, given its established role as a predictor of SA. Additional exploratory analyses evaluated the predictive performance of clinical predictors alone for SA and NSSI, respectively. Furthermore, exploratory sensitivity analyses evaluated the impact of including male participants.

## Materials and methods

### Participants and procedure

Participants were enrolled in a cohort study conducted at the outpatient clinic for risk-taking and self-harming behavior in adolescents (AtR!Sk; Ambulanz für Risikoverhalten und Selbstschädigung) at the Clinic for Child and Adolescent Psychiatry, University Hospital of Heidelberg (ethical approval: S-449/2013). Following clinical diagnosis by trained clinicians, eligible participants were invited to the neurobiological baseline assessment (AtR!Sk-Bio study; ethical approval: S-514/2015). Assessments were conducted at baseline (T0), one year later (T1), and two years later (T2), over the years 2016 to 2021. The main analyses were restricted to female adolescents aged 12–17 years who were proficient in German, reported ≥ 5 NSSI episodes in the year before baseline, and participated in the neurobiological study. This focus on females was chosen because the AtR!Sk-Bio cohort was predominantly female (reflecting the higher proportion of female adolescents presenting at the AtR!Sk outpatient clinic) and restriction to one sex reduced sample heterogeneity in the context of a small sample. Male participants were therefore excluded from the primary analyses but were considered in exploratory sensitivity analyses. Exclusion criteria were pregnancy, acute psychosis, and underlying neurological, endocrinological, or cardiovascular diseases. All participants provided written informed consent and received €40 reimbursement for each assessment. At baseline, this comprised €40 for participation in the AtR!Sk-Bio study, with no reimbursement for the clinical assessments, which were part of routine clinical care. At follow-up 1 and 2, the €40 reimbursement was split into €30 for participation in the AtR! Sk-Bio study and €10 for participation in the clinical assessments.

### Clinical assessments

During clinical assessments, demographic data (e.g. sex, age) and potential confounders (medication intake, substance use, smoking) were collected. At baseline, BPD symptoms were assessed with the Structured Clinical Interview for DSM-IV Axis II Personality Disorders (SCID-II) [43], and psychiatric diagnoses with the Mini International Neuropsychiatric Interview for Children and Adolescents (MINI-KID) [44]. SA and NSSI were assessed at baseline using the German version of the Self-Injurious Thoughts and Behaviors Interview (SITBI-G) [45]. NSSI was defined based on Criterion A of the DSM-5 research diagnosis for NSSI disorder. Additional diagnostic components (Criteria B–F) were not formally assessed. However, this approach is consistent with most current studies in the field, which primarily rely on Criterion A to identify individuals with NSSI. All clinical interviews were conducted by trained clinical staff (psychologists, physicians) working in the AtR!Sk outpatient clinic within a standardized diagnostic framework. Clinicians were trained and continuously supervised in the administration of all structured interviews as part of routine quality procedures. The SITBI-G is a semi-structured interview with excellent psychometric properties, including high interrater reliability and validity [45]. Further details on procedures are provided in the study protocol of the AtR!Sk Bio study [46] and in a description of the AtR!Sk outpatient clinic [47]. Primary outcomes were SA occurrence and NSSI remission between T0 and T2. SA was coded as present (SA = 1) if participants reported ≥ 1 suicide attempt at T1 or T2 for the preceding year, and absent (SA = 0) if no attempts were reported at either time point. For NSSI remission, we assessed three intervals: six months before T1, six months following T1, and six months prior to T2. Participants who reported no NSSI episodes in any of these intervals were considered in remission (NSSI remission = 1), whereas those with at least one episode in any interval were classified as non-remitted (NSSI remission = 0). Pre-baseline SA (i.e., suicide attempts prior to baseline) and NSSI frequency (i.e. NSSI one year prior to baseline) were also assessed at baseline and included as predictors in the analyses.

### Biological measures

Eighteen biomarkers – selected based on their theoretical and clinical relevance to self-harm – were assessed in the overall study at T0, T1 and T2: cortisol (blood), ACTH, C-reactive protein (CRP), interleukin-6 (Il-6), leukocytes, beta-endorphin, oxytocin, estradiol, testosterone, DHEA-S, TSH, fT4, fT3, adrenaline, noradrenaline, dopamine, rHRV (Root Mean Square of Successive Differences; rMSSD), and pain threshold. For prediction analyses, baseline pain threshold, rHRV and four blood markers (TSH, fT3, ACTH, DHEA-S) were selected based on temporal stability. Testosterone was excluded due to collinearity with DHEA-S to ensure variable independence. The selected blood markers demonstrated high retest stability across time points (see supplemental material Table S1 for an overview of retest stability for all biomarkers). Following the guideline of one predictor per 10 cases [48], the number of biological predictors was limited to six in our sample (*n* = 63), comprising rHRV, pain threshold, and four stable biomarkers (see Table 1), to preserve model validity and prevent overfitting.

**Table 1:** Biological markers

Biological System	Biological marker	Measurement
ANS	rHRV	ECG
HPA axis	ACTH	Blood sample
HPG axis	DHEA-S	Blood sample
HPT axis	fT3	Blood sample
HPT axis	TSH	Blood sample
Pain processing	Pain-threshold	Heat plate

ANS, autonomic nervous system; ECG, electrocardiography; rHRV, resting heart rate variability; HPA axis, hypothalamic–pituitary–adrenal axis; HPG axis, hypothalamic–pituitary–gonadal axis; HPT axis, hypothalamic–pituitary–thyroid axis; ACTH, adrenocorticotropic hormone; DHEA-S, dehydroepiandrosterone sulfate; fT3, free triiodothyronine; TSH, thyroid-stimulating hormone

#### Blood samples

After an overnight fast, peripheral blood samples were collected between 8:30 and 9:00 a.m. to control for diurnal hormone variation. Analyses were conducted using accredited procedures at the central laboratories of the Heidelberg University Hospital, Germany. TSH and fT3 were measured via an ADVIA Centaur immunoassay, DHEA-S by an in-house radioimmunoassay, and ACTH in EDTA plasma using a chemiluminescence immunoassay. Reference ranges were: DHEA: 1,3–4 µg/ml; TSH: 0.4–4.0 mU/l; fT3: 2.0–4.2 ng/l; ACTH: 122–466 pg/ml.

#### Resting heart rate variability

rHRV, reflecting autonomic regulation of cardiac functioning, was derived from inter-beat intervals (IBIs), the time between consecutive heartbeats. ECG signals were recorded during a 5-minute resting period at a sampling rate of 1024 Hz using the ECG Move III device (movisens GmbH, Karlsruhe, Germany) as participants completed a standardized color detection task [49] to establish baseline rHRV. Preprocessing included artifact detection and manual R-peak correction using Kubios HRV 3.0 Premium [50], with IBI detrending performed using a smoothing parameter (λ = 500). rHRV analysis was conducted in Stata (Version 18; StataCorp LP, College Station, TX, USA), with the rMSSD (in ms) as the primary metric.

#### Pain threshold

Pain sensitivity was assessed using a TECA Corp. AHP-1800CPV Versatile Cold/Hot Plate (Chicago, IL, USA) with a programmed temperature sequence. Adolescents were instructed to rest their non-dominant hand on the plate. A 3-minute phase for adaptation preceded a gradual temperature increase from 32 °C to 50 °C, achieved over 4 min, with a consistent rise of 1 °C every 13.3 s. Participants were instructed to keep their hand on the plate until the pain became intolerable. The pain threshold was recorded as the temperature (°C) at which pain was first perceived.

### Statistical analyses

Potential confounders were explored by comparing their distribution across outcome groups using chi-square tests and independent samples t-tests. Given the limited sample size and small number of SA cases, additional covariates were not included in the final models to avoid unnecessary model complexity and overfitting.

We used ML to predict SA and NSSI remissions from neurobiological markers assessed at T0. Missing predictor values were imputed via multiple imputation by chained equations [51] using predictive mean matching with 5 nearest neighbors, generating 100 complete datasets. The imputation model included SA, NSSI remissions, TSH, fT3, ACTH, DHEA-S, rHRV, pain threshold, past SA, BPD, operationalized as the number of diagnostic criteria fulfilled (range: 0–9), and age. Imputation was restricted to missing predictor values within the analytical sample and was not used to account for participant dropout or missing outcome data.

To ensure robustness, 100 imputed datasets were generated and analyzed separately. Results were pooled using Rubin’s rules [52] to obtain combined estimates and adjusted standard errors. Initially, TSH, fT3, DHEA-S, ACTH, rHRV, and pain threshold were used as predictors. Number of BPD criteria, past SA or frequency of NSSI within the past 12 months (depending on the outcome), and age were added in the next step. The final model combined predictors from both steps. Outcomes were SA and NSSI remission (yes/no) during the two-years follow-up period. For each phase, three models were applied: linear regression, elastic net regression, and support vector machines (SVM). Exploratory sensitivity analyses were conducted including the male participants (*n* = 8) who met the remaining eligibility criteria to assess whether the restriction to females affected model performance. To assess potential bias due to missing data and dropout, baseline characteristics of participants who completed at least one follow-up assessment were compared with those who dropped out after baseline. Group differences were examined using independent samples t-tests and chi-square tests across demographic, clinical, neurobiological, and behavioral variables.

The models applied represent supervised learning approaches [53]. Logistic regression was included as a baseline model to evaluate whether more complex ML models provide incremental predictive value over a simple and well-established method. Elastic net regression extends logistic regression by adding a regularization term that enables variable selection and coefficient shrinkage, enhancing generalizability to unseen data. This method was selected to address the increased risk of overfitting given the relatively small sample size and number of predictors. SVM offers a flexible framework for classification and regression, particularly effective in high-dimensional settings. By using kernel functions [53], SVMs can model complex, non-linear relationships, making them well-suited for capturing patterns that linear models may miss. A linear SVM was included to explore whether a more flexible algorithm could improve predictive performance beyond linear models, while still being appropriate for small sample sizes. More complex approaches (e.g., tree-based models or neural networks) were not considered given the limited sample size, as such methods typically require larger datasets to avoid overfitting and to ensure stable and generalizable estimates.

To avoid overestimating of model performance, we used repeated cross-validation for internal validation of the models [54, 55]. Each model underwent 20 repetitions of 5-fold cross-validation to ensure robustness. In this approach, the data are repeatedly partitioned into training and validation sets, allowing all observations to contribute to both model training and performance evaluation. This procedure provides a robust estimate of out-of-sample performance, particularly in small samples where a separate hold-out test set would substantially reduce statistical power. Model performance was primarily evaluated using the area under the Receiver operating characteristic (ROC) curve (AUC), which reflects overall discriminative ability. Additional metrics included Accuracy, Brier Score, Positive Predictive Value (PPV), Negative Predictive Value (NPV), Sensitivity, and Specificity, providing information on classification accuracy, probability calibration, and the ability to detect true positives and negatives. Differences in model performance were interpreted descriptively based on these metrics.

All models were fitted and evaluated using R (version 4.3.0; www.r-project.org). Cross-validation and SVM fitting were performed with the *caret* package [version 6.0.94; Kuhn et al. 56], while elastic net regression models were fitted using the *glmnet* package [version 4.1.7; Friedman et al. 57].

Feature importance was evaluated by identifying the most important predictor across 100 imputed datasets for each model. Importance was determined by the absolute value of model coefficients, with the top-ranked predictor in each dataset recorded. The frequency with which each feature ranked first served as an indicator of its stability and importance. Additionally, pooled odds ratios and 95% confidence intervals were estimated using logistic regression in Stata (Version 18; StataCorp LP, College Station, TX, USA) to summarize effect sizes across imputations. The analyses conducted in this study were not preregistered.

## Results

### Descriptive statistics

Of the *n* = 242 adolescents enrolled in the AtR!Sk-Bio study, *n* = 85 attended at least one follow-up (T1 and/or T2). Thirteen individuals not meeting Criterion A of the DSM-5 research diagnosis for NSSI disorder, eight males, and one participant outside the age range were excluded. The final sample comprised *n* = 63 female adolescents (*n* = 12 with SA and *n* = 51 without SA during the follow-up period; *n* = 31 with NSSI remission and *n* = 32 without NSSI remission during the follow-up period, see Table 2 for an overview of sample characteristics). Of these, *n* = 62 participated at T1 and *n* = 37 at T2. To assess potential dropout bias, participants who completed at least one follow-up were compared with those who dropped out after baseline across baseline variables (see supplemental material Table S2). A small difference was found for smoking, with higher rates among participants who dropped out (61.2%) compared to those who remained in the study (42.9%; *χ*²(1) = 4.50, *p* = 0.034, Cramér’s *V* = 0.18), but no consistent pattern across related variables emerged.

**Table 2:** Sample characteristics for adolescents with and without SA during the follow-up period

	Overall (*N* = 63)	No SA (*N* = 51)	SA (*N* = 12)	Statistics
NSSI-Remission; *n* (%)	15.1 (1.45)	27 (52.9%)	4 (33.3%)	χ²_(1, *N* = 63)_ = 0.81, *p* = 0.367. Cramér’s *V* = 0.15.
Age; Mean (SD)	15.0 [12.0, 17.0]	15.1 (1.50)	14.8 (1.22)	*t*_(20)_ = 0.95, *p* = 0.36
Past Suicide Attempt; n (%)	25 (39.7%)	16 (31.4%)	9 (75.0%)	χ²_(1, *N* = 63)_ = 6.01, *p* = 0.014. Cramér’s *V* = 0.35.
Past NSSI; Mean (SD)	79.2 (81.7)	75.4 (79.1)	95.5 (93.9)	*t*_(15)_ = − 0.69, *p* = 0.502, *d* = − 0.25
Psychoactive medication; n (%)	3 (4.8%)	3 (5.9%)	0 (0%)	*χ²*_*(*1, *N* = 63)_ = 0.01, *p* = 0.914. Cramér’s *V* = 0.11
Drug consumption; n (%)	15 (23.8%)	11 (21.6%)	4 (33.3%)	*χ²*_(1, *N* = 63)_ = 0.23, *p* = 0.628. Cramér’s *V* = 0.11
Smoking; n (%)	27 (42.9%)	22 (43.1%)	5 (41.7%)	*χ²*_(1, *N* = 63)_ = 0, *p* = 1. Cramér’s *V* = 0.01.
BPD Symptoms; Mean (SD)	3.17 (2.10)	2.76 (1.93)	4.92 (1.93)	*t*_(17)_ = − 3.48, *p* = 0.003, *d* = − 1.11
ACTH; Mean (SD)	17.5 (9.65)	17.9 (9.50)	15.4 (10.6)	*t*_(12)_ = 0.68, *p* = 0.51, *d* = 0.25
TSH; Mean (SD)	2.22 (1.32)	2.41 (1.38)	1.42 (0.523)	*t*_(44)_ = 3.88, *p* < 0.001, *d* = 0.78
fT3; Mean (SD)	3.43 (0.410)	3.48 (0.397)	3.25 (0.433)	*t*_(14)_ = 1.6, *p* = 0.133, *d* = 0.5
DHEA-S; Mean (SD)	1.89 (1.10)	1.96 (1.12)	1.57 (0.951)	*t*_(15)_ = 1.15, *p* = 0.269, *d* = 0.36
rHRV (rMSSD); Mean (SD)	49.3 (23.6)	49.5 (24.2)	48.5 (21.7)	*t*_(23)_ = 2.21, *p* = 0.038, *d* = 0.58
Pain Threshold; Mean (SD)	42.7 (3.89)	42.2 (4.0)	44.5 (2.83)	*t*_*(*23)_ = -2.33, *p* = 0.029, *d* = -0.61
F10-F19, n (%)	9 (14.3%)	7 (13.7)	2 (16.6)	*OR* = 1.25, 95% *CI*[0.11, 8.08], *p* = 1
F30-F39, n (%)	40 (63.5%)	32 (62.7)	8 (66.7)	*OR* = 1.18, 95% *CI*[0.27, 6.12], *p* = 1
F40-F48, n (%)	26 (41.3%)	21 (41.2)	5 (41.7)	*OR* = 1.02, 95% *CI*[0.22, 4.34], *p* = 1
F50-F59, n (%)	8 (12.7%)	6 (11.8)	2 (16.7)	*OR* = 1.49, 95% *CI*[0.13, 10.11], *p* = 1
F60-F69, n (%)	24 (38.1%)	15 (29.4)	9 (75)	-
F90-F98, n (%)	12 (19.0%)	8 (15.6)	4 (33.3)	*OR* = 2.64, 95% *CI*[0.47, 13.20], *p* = 0.22

SA = Suicide attempt; SD = standard deviation, n = number of participants; Statistical tests refer to comparisons between the SA and no SA groups; Smoking: on more than one day in the last year. Drug consumption: at least one day of drug consumption during the last year. fT3 = free triiodothyronine, ACTH = adrenocorticotropic hormone, BPD = Borderline Personality Disorder, DHEA-S = dehydroepiandrosterone-sulfate, rHRV = resting heart rate variability, TSH = thyroid stimulating hormone, NSSI = non-suicidal self-injury. Case numbers vary due to missing data

no F0, F2, F7 or F8 disorders were diagnosed. For F6, only BPD was diagnosed

Chi-square tests of independence and independent sample *t-tests* were used to examine associations between psychiatric diagnoses and potential confounders (medication intake, substance use, smoking) across outcome groups (see Table 2). Adolescents with past SA had significantly more BPD symptoms (*M* = 4.52, *SD* = 1.90) compared to those without past SA (*M* = 2.29, *SD* = 1.74, *t*(48) = − 4.72, *p* < 0.001, *d* = − 1.24). Likewise, adolescents with a SA during the follow-up period showed more BPD symptoms at baseline (*M* = 4.92, *SD* = 1.93) than those without SA during this period (*M* = 2.76, *SD* = 1.93, *t*(17) = − 3.48, *p* = 0.003, *d* = − 1.11).

### Association between SA and biomarkers

**Fig. 1:**
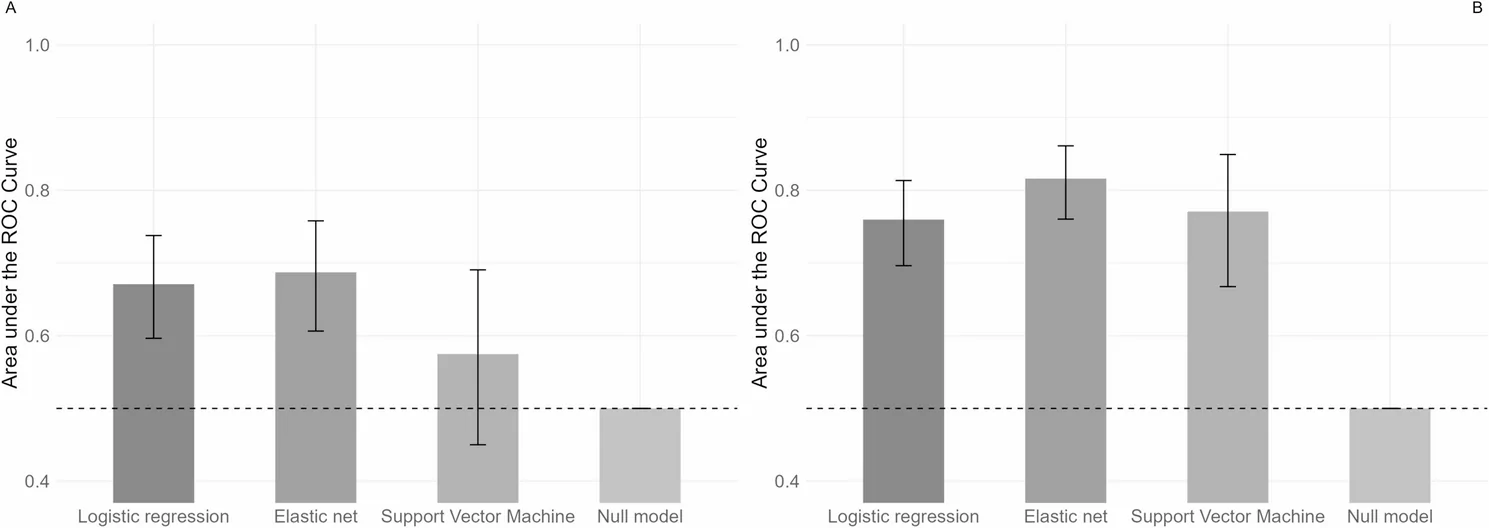
AUC for models predicting future SA using the 6 defined biomarkers (**A**) before and (**B**) after including BPD symptoms, past SA, and age as covariates. The bars represent the performance of machine learning models: A model with AUC = 1 would be considered as perfect, 0.9 < AUC < 1 as excellent, 0.8 < AUC < 0.9 as good, 07 < AUC < 0.8 as fair, 0.6 < AUC < 0.7 as poor and 0.5 < AUC < 0.6 as being slightly better than chance. The error bars represent the 95% confidence intervals (CIs) of AUC values

ML models using the six selected biomarkers showed poor to moderate performance in predicting SA (Fig. 1A; Table 3). Logistic regression and elastic net regression showed higher AUC values than SVM. For these models, the estimated confidence intervals of the AUC were predominantly above chance level (AUC = 0.5), whereas SVM performance was at or near chance level. The models exhibited strong performance in correctly identifying negative cases, with excellent specificity, but performed poorly in detecting positive cases, as indicated by low sensitivity. In the logistic regression model, low TSH showed a non-significant trend toward predicting SA (OR = 0.34, 95% CI = 0.03–1.20 without covariates, see supplemental material Table S3; OR = 0.35, 95% CI = 0.06–2.10 with covariates; ). fT3 consistently emerged as the most important biomarker across all ML models. Including male participants did not meaningfully alter the results.

**Table 3:** Pooled metrics, standard deviation, and confidence intervals for models predicting SA using the defined 6 biomarkers

Biomarkers only	Metric	Pooled	SD	LB	UB
Logistic Regression	AUC	0.671	0.162	0.596	0.738
	Accuracy	0.747	0.014	0.717	0.774
	Brier Score	0.176	0.007	0.163	0.187
	Sensitivity	0.111	0.075	0.027	0.363
	Specificity	0.894	0.015	0.859	0.921
	PPV	0.196	0.101	0.064	0.466
	NPV	0.812	0.011	0.789	0.834
Elastic Net Regression	AUC	0.687	0.179	0.606	0.758
	Accuracy	0.785	0.035	0.707	0.847
	Brier Score	0.156	0.011	0.141	0.180
	Sensitivity	0.008	0.040	0.001	0.992
	Specificity	0.987	0.047	0.049	0.999
	PPV	0.115	0.819	0.001	0.999
	NPV	0.811	0.008	0.794	0.827
Support Vector Machine	AUC	0.575	0.253	0.450	0.691
	Accuracy	0.772	0.016	0.737	0.802
	Brier Score	0.155	0.001	0.153	0.157
	Sensitivity	0.024	0.057	0.001	0.756
	Specificity	0.947	0.036	0.813	0.987
	PPV	0.087	0.203	0.001	0.939
	NPV	0.807	0.005	0.796	0.818
Biomarkers + clinical features	Metric	Pooled	SD	LB	UB
Logistic Regression	AUC	0.760	0.162	0.696	0.813
	Accuracy	0.790	0.017	0.754	0.822
	Brier Score	0.172	0.007	0.158	0.187
	Sensitivity	0.410	0.073	0.275	0.559
	Specificity	0.879	0.012	0.853	0.901
	PPV	0.442	0.052	0.343	0.546
	NPV	0.864	0.014	0.833	0.890
Elastic Net Regression	AUC	0.816	0.169	0.760	0.861
	Accuracy	0.803	0.011	0.781	0.824
	Brier Score	0.132	0.005	0.123	0.142
	Sensitivity	0.015	0.079	0.000	0.999
	Specificity	0.976	0.032	0.740	0.998
	PPV	0.059	0.349	0.000	1.000
	NPV	0.822	0.023	0.773	0.863
Support Vector Machine	AUC	0.771	0.260	0.668	0.849
	Accuracy	0.786	0.020	0.743	0.823
	Brier Score	0.146	0.005	0.139	0.157
	Sensitivity	0.154	0.483	0.000	0.997
	Specificity	0.928	0.109	0.335	0.997
	PPV	0.243	0.692	0.000	0.998
	NPV	0.841	0.024	0.787	0.884

Covariates in the predictive models include past SA, BPD symptoms, and age. Reported performance metrics include ROC (area under the receiver operating characteristic curve), Accuracy, Brier Score (mean squared difference between predicted probabilities and actual outcomes), Sensitivity (true positive rate), Specificity (true negative rate), PPV (positive predictive value), and NPV (negative predictive value). A model with AUC = 1 is considered perfect, 0.9 < AUC ≤ 1 as excellent, 0.8 < AUC ≤ 0.9 as good, 0.7 < AUC ≤ 0.8 as fair, 0.6 < AUC ≤ 0.7 as poor, and 0.5 < AUC ≤ 0.6 as slightly better than chance. LB and UB refer to the lower and upper bounds of the 95% confidence intervals (CIs), respectively

### Association between SA, biomarkers, BPD, past SA, and age

Models incorporating the six biomarkers, BPD, and past SA demonstrated moderate predictive performance for future SA (Fig. 1B; Table 3). Logistic regression and elastic net regression showed higher AUC values than SVM. For these models, the estimated confidence intervals of the AUC were predominantly above chance level (AUC = 0.5), whereas SVM performance was at or near chance level. Again, the models demonstrated strong performance in predicting negative cases, with excellent specificity; however, their performance in identifying positive cases was limited, as reflected by low sensitivity. Past SA was the strongest predictor across all ML models, with an odds ratio of 7.28 (95% CI = 0.80–66.68; see supplemental material Table S4). Including male participants did not meaningfully alter the results.

### Association between SA, BPD, past SA, and age

ML models including BPD and age showed moderate performance in predicting SA (see supplemental material Figure S1, Table S5). Logistic regression and elastic net regression showed higher AUC values than SVM. For these models, the estimated confidence intervals of the AUC were predominantly above chance level (AUC = 0.5), whereas SVM performance was at or near chance level. The models demonstrated good performance in predicting negative cases, with excellent specificity, and moderate performance in predicting positive cases; however, sensitivity remained poor. For SVM, the inclusion of past SA improved predictive performance. However, for logistic regression and elastic net regression performance remained stable (see supplemental Figure S2, Table S6). Logistic regression and elastic net achieved higher overall accuracy and lower Brier scores, while SVM showed a notable increase in AUC and sensitivity. The models demonstrated good performance in predicting negative cases, with excellent specificity, and improved performance in predicting positive cases. Sensitivity, although higher after including past SA, remained limited. Including male participants did not meaningfully alter the results.

### Association between NSSI remission and biomarkers

**Fig. 2:**
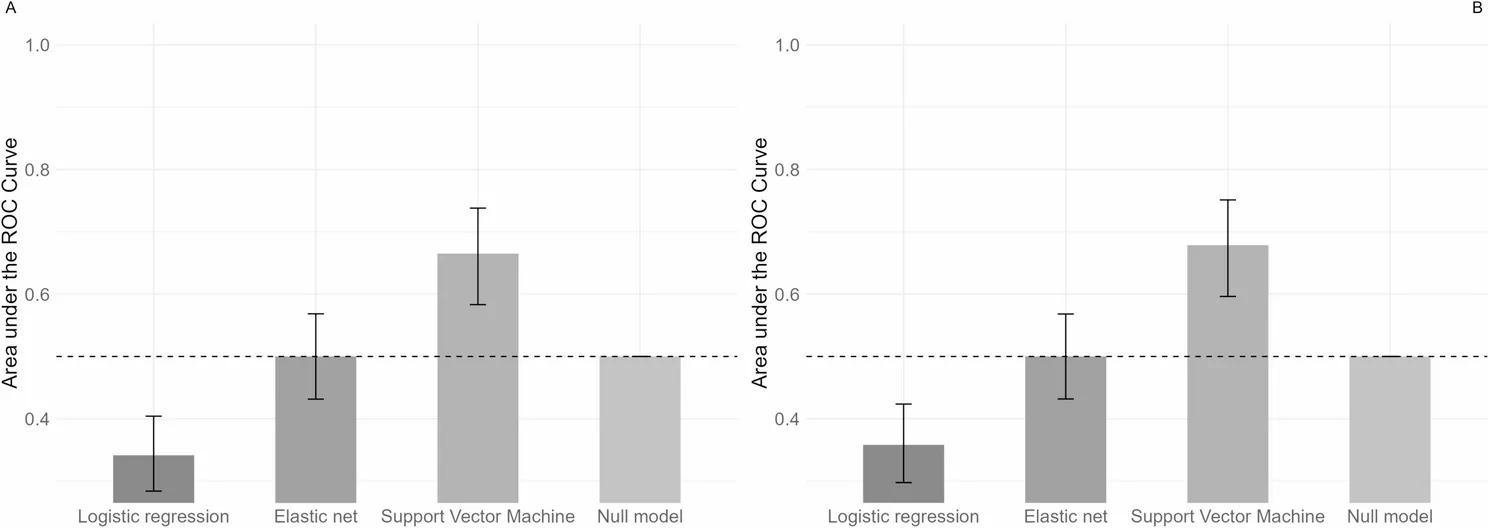
AUC for models predicting NSSI remissions using the 6 defined biomarkers (**A**) before and (**B**) after including past SA, BPD, and age as covariates. Notes: The bars represent the performance of machine learning models: A model with AUC = 1 would be considered as perfect, 0.9 < AUC < 1 as excellent, 0.8 < AUC < 0.9 as good, 07 < AUC < 0.8 as fair, 0.6 < AUC < 0.7 as poor and 0.5 < AUC < 0.6 as being slightly better than chance. The error bars represent the 95% confidence intervals (CIs) of AUC values

ML models using the six biomarkers demonstrated poor performance in predicting NSSI remission (Fig. 2A; Table 4). Logistic regression performed below chance (AUC = 0.34), suggesting possible overfitting to noise. Elastic net regression showed no predictive ability beyond chance, while SVM performed slightly better but remained poor. All models exhibited poor predictive performance for both negative and positive cases, with low specificity and sensitivity. Including male participants did not meaningfully alter the results.

**Table 4:** Pooled metrics, standard deviation and confidence intervals for models predicting NSSI remission using the 6 defined biomarkers

Biomarkers only	Metric	Pooled	SD	LB	UB
Logistic Regression	AUC	0.342	0.135	0.284	0.404
	Accuracy	0.384	0.038	0.312	0.461
	Brier Score	0.306	0.005	0.296	0.315
	Sensitivity	0.359	0.047	0.272	0.457
	Specificity	0.408	0.039	0.334	0.486
	PPV	0.370	0.041	0.293	0.454
	NPV	0.396	0.035	0.329	0.468
Elastic Net Regression	AUC	0.500	0.139	0.432	0.568
	Accuracy	0.484	0.002	0.480	0.489
	Brier Score	0.250	0.000	0.250	0.250
	Sensitivity	0.287	0.029	0.233	0.349
	Specificity	0.675	0.033	0.606	0.737
	PPV	0.462	0.000	0.462	0.462
	NPV	0.494	0.002	0.490	0.498
Support Vector Machine	AUC	0.665	0.176	0.583	0.738
	Accuracy	0.383	0.036	0.314	0.457
	Brier Score	0.239	0.008	0.226	0.253
	Sensitivity	0.292	0.094	0.143	0.503
	Specificity	0.467	0.124	0.246	0.703
	PPV	0.348	0.040	0.273	0.431
	NPV	0.404	0.040	0.328	0.485
Biomarkers + clinical features	Metric	Pooled	SD	LB	UB
Logistic Regression	AUC	0.358	0.139	0.298	0.424
	Accuracy	0.402	0.033	0.338	0.469
	Brier Score	0.333	0.006	0.321	0.346
	Sensitivity	0.384	0.041	0.306	0.468
	Specificity	0.419	0.037	0.348	0.494
	PPV	0.390	0.035	0.323	0.462
	NPV	0.413	0.032	0.351	0.478
Elastic Net Regression	AUC	0.500	0.138	0.432	0.568
	Accuracy	0.484	0.003	0.479	0.489
	Brier Score	0.250	0.000	0.250	0.250
	Sensitivity	0.288	0.032	0.230	0.355
	Specificity	0.674	0.036	0.600	0.740
	PPV	0.462	0.000	0.462	0.462
	NPV	0.494	0.002	0.490	0.499
Support Vector Machine	AUC	0.679	0.180	0.596	0.751
	Accuracy	0.384	0.029	0.328	0.443
	Brier Score	0.233	0.007	0.221	0.247
	Sensitivity	0.260	0.088	0.125	0.465
	Specificity	0.500	0.103	0.307	0.693
	PPV	0.335	0.045	0.252	0.431
	NPV	0.411	0.030	0.353	0.471

Covariates in the predictive models include a history of nonsuicidal self-injury (NSSI) before baseline, BPD symptoms, and age. Reported performance metrics include ROC (area under the receiver operating characteristic curve), Accuracy, Brier Score (mean squared difference between predicted probabilities and actual outcomes), Sensitivity (true positive rate), Specificity (true negative rate), PPV (positive predictive value), and NPV (negative predictive value). A model with AUC = 1 is considered perfect, 0.9 < AUC ≤ 1 as excellent, 0.8 < AUC ≤ 0.9 as good, 0.7 < AUC ≤ 0.8 as fair, 0.6 < AUC ≤ 0.7 as poor, and 0.5 < AUC ≤ 0.6 as slightly better than chance. LB and UB refer to the lower and upper bounds of the 95% confidence intervals (CIs), respectively

### Association between NSSI remission, biomarkers, BPD, past NSSI, and age

ML models including the six biomarkers, BPD, past NSSI, and age showed poor performance in predicting NSSI remissions (Fig. 2B; Table 4). Logistic regression performed below chance (AUC = 0.36), indicating possible overfitting to noise. Elastic net regression showed no predictive ability beyond chance, while SVM performed slightly better but remained poor. Again, all models demonstrated poor performance in terms of both negative and positive predictive values, as well as low specificity and sensitivity. Including males in the analyses did not significantly alter results.

### Association between NSSI remission, BPD, past NSSI, and age

ML models including BPD, past NSSI, and age showed poor performance in predicting NSSI remissions (see supplemental material Figure S3, Table S7). Logistic regression performed below chance (AUC = 0.40), possibly due to overfitting to noise. Elastic net regression showed no predictive ability beyond chance, and SVM performed slightly better but remained poor. Once again, all models performed poorly across key diagnostic metrics, including sensitivity, specificity and both positive and negative predictive values. Including male participants did not meaningfully alter the results.

## Discussion

In this sample of adolescents with NSSI, ML models showed moderate performance in predicting SA over two years based on biomarkers of the neuroendocrine and autonomic nervous systems, as well as pain sensitivity. Logistic and elastic net regression outperformed the more complex SVM. Prediction improved substantially when clinical variables and age were added, with the slightly best performance achieved using only past SA, BPD, and age. Past SA was the strongest predictor, followed by TSH and fT3. Despite the large effect, the prediction by past SA is not significant, presumably due to its high covariance with BPD. In logistic regression, lower TSH and fT3 levels showed non-significant trends towards associations with future SA. In contrast, ML models did not predict NSSI remission over chance level.

In contrast to prior cross-sectional findings reporting superior performance of complex over simpler ML models in identifying adolescents with lifetime SA [23], our longitudinal findings indicate that logistic regression performed comparably to elastic net regression and outperformed SVM. Comparing models with (a) biomarkers only, (b) biomarkers plus clinical variables and age, and (c) clinical variables and age alone revealed no added value of biomarkers. This contrasts with Videtič and Kouter’s [17] call for ML-based biomarker screening and Zhang et al.’s [16] finding that physiological factors improve SA prediction in adults. Consistently, behavior-related predictors – especially mental health and lifestyle factors – remain the strongest predictors. Ehtemam et al. [13] reported good ML prediction including biological variables, but the low proportion of such studies (< 6%) limits generalizability. Schafer et al. [15] found that ML models significantly outperformed traditional theory-driven models in predicting suicidal ideation, SA and suicide. However, within the theory-driven models, those based on biological theories showed the least accuracy in predicting suicidality.

The greater predictive power of clinical over biological variables for suicidality may have several explanations. First, past SA – the strongest predictor in our models – is a direct precursor of future SA and likely is confounded with related factors such as lifestyle [16], stressful life events or personality traits [58]. Second, although biomarkers were chosen for temporal stability, their correlations varied across the 2-year follow-up, indicating substantial fluctuations, unlike the stable history of SA and BPD. Biomarkers may predict better as proximal indicators over shorter time frames (e.g. within ecological momentary assessment, EMA). Third, biological suicide risk profiling is still nascent [17], whereas clinical and demographic predictors have been extensively studied and more precisely selected based on prior evidence [10].

Finally, the stress-diathesis model suggests that suicide may result from the interaction of environmental stressors and a biologically-driven susceptibility [59]. It is conceivable, that past SA, the strongest predictor of future SA in our models, could already reflect this biological vulnerability. In this case, controlling for past SA, may have accounted for the variance related to potential biomarker influences. This hypothesis is supported by our previous study, which found significant associations between a set of biomarkers and past SA, based on the baseline data of the current sample [23]. However, it should be critically noted that a potential biological predisposition to suicidal behavior becomes clinically relevant only through longitudinal prediction. The costly and time-consuming determination of biomarkers would not be justified in clinical practice if the additional information provided is not greater than what is already available from assessing past SA.

Importantly, all our models predicting SA demonstrated good to excellent negative predictive performance (NPV) and specificity but poor positive predictive performance (PPV) and sensitivity. In other words, the models accurately identified adolescents without SA during follow-up but poorly detected those who engaged in SA. This discrepancy likely reflects the unequal class distribution in our sample (*n* = 12 with SA vs. *n* = 51 without), which may have inflated NPV and suppressed PPV. Clinically, however, the critical goal is to accurately identify adolescents at risk for SA, highlighting a limitation of the current models.

In general, past SA, as the strongest predictor for future SA highlights the need for evidence-based suicide prevention programs targeting adolescents after suicide attempts. Kennard et al. [60] emphasized the need for effective, low-cost outpatient programs as alternatives or supplement to brief inpatient care. Their 4–6 week intensive outpatient group therapy showed a reduction in depressive symptoms and suicidal ideation, although the study lacked a control group for comparison. In contrast, Asarnow et al. [61] demonstrated in a randomized controlled trial that the cognitive-behavioral, family-centered SAFETY program was superior to enhanced treatment as usual in reducing SA over a 3-month follow-up interval.

Regarding the predictive value of biomarkers, fT3 and TSH emerged as the most prominent biological predictors within our ML models and showed a non-significant trend toward a negative association with SA. However, the variable importance of both markers declined substantially when past SA was included as predictors. This suggests that biomarkers, particularly those related to HPT axis functioning, may reflect a general vulnerability to SA, but do not specifically predict future SA. Previous research has linked autoimmune thyroiditis and both subclinical or overt hypothyroidism to depression and anxiety disorders in adults [62]. Additionally, negative associations of both fT3 and TSH with borderline personality disorder severity and symptomatic distress have been reported in adolescents engaging in NSSI [35]. While evidence regarding TSH alterations in adults with a history of SA remains inconclusive [29, 36] – possibly reflecting a complex interplay between TSH, fT3 and fT4 – meta-analytic findings, consistent with the results of the present study, support a negative association between fT3 levels and SA in adults [36]. Alterations in HPT axis function may contribute to SA indirectly by affecting neurotransmitter systems, neuroplasticity, and the functioning of brain regions involved in emotion regulation and cognitive control [63]. However, numerous additional factors, such as social isolation, hopelessness, and impulsivity, also play a role in the development of suicidal ideation and its progression, potentially attenuating the impact of HPT axis dysfunction.

Another aim of this study was to apply ML models to predict the two-year course of NSSI remission. However, the models did not consistently outperform chance level, challenging findings by Mürner-Lavanchy et al. [22], who reported successful discrimination between adolescents with and without NSSI disorder using biomarkers and ML. This discrepancy may be due to methodological differences: while Mürner-Lavanchy et al. [22] employed a cross-sectional design to classify diagnostic status, we used a longitudinal approach to predict NSSI outcomes within a clinical sample already diagnosed with NSSI.

The lack of predictive performance in our study may be attributed to the long follow-up interval and substantial variability in NSSI remission. Prior research in both adolescent population-based [64] and clinical samples [65] has shown that NSSI can fluctuate over short periods, with distinct trajectory classes emerging over time. Supporting this, our group previously found that 75% of adolescents engaging in NSSI reduced their frequency by at least 50% within one year [24]; however, only one-third of responders achieved full remission, and 41% of those relapsed within another year. The operationalization of SA and NSSI as dichotomous outcomes may have resulted in an oversimplification of complex behaviors with dynamic trajectories. Moreover, adolescence is a developmental period marked by symptom shifts, including transitions from NSSI to substance use [66, 67].

Taken together, our ML models may have failed to predict long-term NSSI remission due to short-term fluctuations in behavior, high rates of partial response or relapse, and potential undetected symptom shifts. A promising approach to capturing short-term fluctuations in NSSI is the integration of biomarker assessments with EMA. The method of EMA provides detailed, real-time insights into the mechanisms underlying NSSI urges, thoughts, and behaviors. Evidence suggests that individuals engaging in NSSI exhibit greater instability across cognitive, interpersonal, and affective domains [68–70], as well as increases in negative cognitions and emotions immediately preceding self-injury [26, 71, 72].

Recent work combining EMA with cardiac autonomic measures found that decreases in heart rate (HR) and increases in HRV precede NSSI urges, indicating a potentially attenuated sympathetic stress response in the hour before these urges occur [73]. Despite these advances, studies integrating EMA with biomarker data remain limited, likely due to the logistical and methodological challenges associated with high-frequency biological sampling. In particular, biomarker collection methods such as blood sampling and pain sensitivity assessments may be too burdensome to capture rapid, day-to-day or week-to-week fluctuations in NSSI dynamics. To sum up, future research should incorporate more frequent follow-up assessments, consider co-occuring trajectories of NSSI and substance use, and explore biomarker-informed ML models to enhance predictive accuracy.

### Strengths and limitations

To our knowledge, this is the first study to longitudinally examine the prediction of SA and NSSI remission using ML in an adolescent high-risk sample. A major strength lies in the integration of both biomarkers and clinical variables within advanced ML frameworks. Clinical assessments of NSSI and SA were conducted by trained clinicians using standardized interviews. Biomarkers were selected based on theoretical relevance and temporal stability, covering neuroendocrine and autonomic systems as well as pain sensitivity.

However, several limitations should be considered when interpreting the findings. First, the sample size of the present study is small with a substantial imbalance between the groups (SA vs. no SA), and a limited number of SA (*n* = 12). Accordingly, this work should be considered as proof-of-concept study, and findings require replication in larger samples. Although, small sample sizes are common in longitudinal clinical and biomarker research, ML approaches can still be applied under such conditions when appropriate methodological constraints are implemented [74]. In the present study, we addressed this limitation by limiting the number of predictors relative to the number of cases, selecting biologically stable markers, and applying regularized models (e.g. elastic net) together with repeated cross-validation to reduce the risk of overfitting and enhance robustness. Furthermore, data quality can be considered moderate to good, which can enhance ML performance in smaller samples [75]. For similar reasons, potentially relevant confounders were not included in the final models in order to maintain model parsimony and reduce overfitting risk [74]. Consequently, residual confounding cannot be fully excluded, and some omitted variables may still hold predictive or confounding value that could not be detected in the present sample. Nevertheless, future research is essential to replicate these preliminary findings in larger and more balanced samples. Second, inclusion was limited to participants with at least one follow-up assessment, resulting in *n* = 1 dropout for T1 and *n* = 26 dropouts for T2. The higher dropout rate of T2 is particularly concerning, as it may have led to misclassification of some participants into the “no SA” group due to missing follow-up data. Although dropout analyses did not reveal systematic differences between participants who remained in the study and those who dropped out after baseline, selective attrition cannot be fully excluded and may limit generalizability. In addition, although structured interviews were conducted by trained and supervised clinical staff within a standardized diagnostic framework, interrater reliability was not formally assessed within the present study, which limits the ability to directly evaluate diagnostic consistency. Third, our primary outcomes were defined dichotomous (SA: yes/no; NSSI remission: yes/no), which did not capture variations in severity (e.g., repetition, lethality, medical treatment needs) or interim remissions of NSSI between the annual assessments. Finally, although biomarkers were chosen based on stability, test-retest reliability varied substantially, especially for ACTH, fT3, and TSH. Predictive utility of biomarkers may improve by examining proximal biological traits over shorter time intervals. Moreover, although biomarkers were initially selected based on theoretical relevance, the subset included in the prediction models was ultimately chosen for temporal stability to limit the number of predictors and reduce the risk of overfitting given the small sample size. This approach may have led to the exclusion of biologically relevant markers that could have contributed to prediction. In addition, the selected biomarkers themselves exhibited fluctuating stability coefficients, suggesting that they may not provide sufficient predictive power as proximal traits. Future studies with larger samples should therefore aim to include a broader range of biomarkers, ideally with more frequent and state-specific measurements, to better capture the multifactorial and dynamic nature of self-harm.

### Summary and conclusions

The present study supports the potential of ML for predicting SA over a two-year period in a high-risk adolescent sample characterized by risk-taking and self-harming behavior. However, combining biological markers – spanning the neuroendocrine (ACTH; TSH, fT3; DHEA-S), and autonomic nervous (rHRV) systems, and pain threshold – with clinical variables (past SA; BPD) and age did not improve predictive performance compared to models including only clinical variables and age. Past SA consistently emerged as the strongest predictor across ML models, highlighting the critical need for targeted interventions following adolescent suicide attempts. However, our models primarily performed well in identifying negative cases but were less effective at detecting positive cases, limiting their clinical utility. Among biological variables, TSH and fT3 were the most prominent predictors, although their association with SA did not reach significance. In contrast, ML models predicting NSSI remission did not consistently exceed chance-level performance. Future studies should explore the predictive utility of proximal biological markers in ML models with more frequent and shorter follow-up intervals.

## Supplementary Information

Below is the link to the electronic supplementary material.

Supplementary Material 1.

## Data Availability

For privacy reasons concerning patient data, the datasets used in this study are not publicly accessible. They may be obtained from the corresponding author upon reasonable request.

## Abbreviations

ACTH: Adrenocorticotropic hormone
ANS: Autonomic nervous system
AUC: Area under the receiver operating characteristic curve
BPD: Borderline personality disorder
CGAS: Children’s Global Assessment Scale
CRP: C-reactive protein
DHEA-S: Dehydroepiandrosterone sulfate
DSM-5: Diagnostic and Statistical Manual of Mental Disorders, 5th edition
ECG: Electrocardiogram
fT3: Free triiodothyronine
fT4: Free thyroxine
HPA axis: Hypothalamic-pituitary-adrenal axis
HPG axis: Hypothalamic-pituitary-gonadal axis
HPT axis: Hypothalamic-pituitary-thyroid axis
HR: Heart rate
HRV / rHRV: Resting heart rate variability
IBI: Inter-beat interval
Il-6: Interleukin-6
MINI-KID: Mini International Neuropsychiatric Interview for Children and Adolescents
ML: Machine learning
NSSI: Non-suicidal self-injury
NPV: Negative predictive value
PPV: Positive predictive value
ROC: Receiver operating characteristic
rMSSD: Root mean square of successive differences (HRV metric)
SA: Suicide attempt
SCID-II: Structured Clinical Interview for DSM-IV Axis II Personality Disorders
SITBI-G: German version of the Self-Injurious Thoughts and Behaviors Interview
SVM: Support vector machine
TSH: Thyroid-stimulating hormone
