# Supplementary material for: Integrating neurobiological markers to prospectively predict adolescent non-suicidal self-injury and suicide attempts: a machine learning approach
Source: Child Adolesc Psychiatry Ment Health. 2026 Jun 22;20:127. doi: 10.1186/s13034-026-01122-4 (PMC13576133; doi:10.1186/s13034-026-01122-4)
Supplement: Supplementary file 1 [file 13034_2026_1122_MOESM1_ESM.docx]

**Supplementary materials**

**Integrating neurobiological markers to prospectively predict adolescent non-suicidal self-injury and suicide attempts: a machine learning approach**

Reichl, C., Fink, E., von den Driesch, L., Lerch, S., Koenig, J., Berger, T., & Kaess, M.

**1. Tables**

Table S1. Pearson correlation coefficients of biomarkers across three time points (Baseline, T1, and T2)

| Biomarker | Baseline X T1 | T1 X T2 | Baseline X T2 |
| --- | --- | --- | --- |
| **DHEA-S** | **0.780**** | **0.870**** | **0.743**** |
| Testosterone | 0.533** | 0.576* | 0.517 |
| **ACTH** | **0.521**** | **0.273** | **0.414** |
| **fT3** | **0.061** | **0.247** | **0.738**** |
| **TSH** | **0.420**** | **0.623**** | **-0.058** |
| Cortisol | 0.142 | 0.551* | 0.200 |
| Noradrenalin | 0.130 | 0.238 | 0.453 |
| Beta-Endorphin | 0.269 | -0.118 | 0.520* |
| Adrenalin | -0.001 | -0.092 | 0.528 |
| fT4 | 0.193 | 0.011 | 0.205 |
| Il-6 | 0.686 | -0.122 | -0.181 |
| Estradiol | 0.114 | -0.131 | 0.230 |
| Leukocytes | 0.210 | -0.037 | -0.102 |
| CRP | 0.066 | 0.566 | -0.127 |
| Oxytocin | -0.070 | Not available | Not available |
| Dopamine | -0.190 | -0.108 | -0.286 |

**Notes:** CRP C-reactive protein, Il-6 Interleukin-6, fT3 free triiodothyronine, fT4 free thyroxine, ACTH adrenocorticotropic hormone, DHEA-S dehydroepiandrosterone-sulfate, TSH thyroid stimulating hormone. For oxytocin only data from baseline and the 1-year follow-up are available.

Table S2. Sample characteristics for female patients with NSSI at baseline

|  | | | **BL and FU (N=63)** | **BL only  (N=98)** | **Statistics** |  |  |
| --- | --- | --- | --- | --- | --- | --- | --- |
| **Age** | | |  |  |  |  |  |
| Mean (SD) | | | 15.1 (1.45) | 14.8 (1.47) | *t*(134.12) = 1.18, *p* = .240, *d* = 0.19 |  |  |
| **BPD-Symptoms** | | |  |  |  |  |  |
| Mean (SD) | | | 3.17 (2.10) | 3.49 (2.06) | *t*(130.44) = –0.94, *p* = .350, *d* = –0.15 |  |  |
|  |  |  |  |  |  |  |  |
| **Depressive symptoms** | | |  |  |  |  |  |
| Mean (SD) | | | 31.1 (8.58) | 29.2 (9.26) | *t*(128.49) = 1.25, *p* = .214, *d* = 0.21 |  |  |
| Missing | | | 5 (7.9%) | 14 (14.3%) |  |  |  |
| **Past suicide attempts** | | |  |  |  |  |  |
|  |  |  |  |  |  |  |  |
| Yes | | | 25 (39.7%) | 52 (53.1%) | *χ²*(1, *N* = 161) = 2.24, *p* = .134. Cramér’s *V* = 0.13 |  |  |
|  |  |  |  |  |  |  |  |
| **Past NSSI** | | |  |  |  |  |  |
| Yes | | | 79.2 (81.7) | 63.0 (67.9) | *t* (1.3082)= 114.89, *p* = 0.193, *d* = 0.22 |  |  |
|  | | |  |  |  |  |  |
| **Psychoactive medication** | | |  |  |  |  |  |
| Yes | | | 3 (4.8%) | 14 (14.3%) | *χ*²(1, *N* = 161) = 2.74, *p* = .098. Cramér’s *V* = 0.15 |  |  |
| **Drug consumption** | | |  |  |  |  |  |
| Yes | | | 15 (23.8%) | 37 (37.8%) | *χ*²(1, *N* = 161) = 2.80, *p* = .094. Cramér’s *V* = 0.15 |  |  |
| **Smoking** | | |  |  |  |  |  |
| Yes | | | 27 (42.9%) | 60 (61.2%) | χ²(1, *N* = 161) = 4.50, *p* = .034. Cramér’s *V* = 0.18 |  |  |
|  |  |  |  |  |  |  |  |
| **F10-F19 diagnoses** | | |  |  |  |  |  |
|  |  |  |  |  |  |  |  |
| Yes | | | 9 (14.3%) | 26 (26.5%) | *χ*²(1, *N* = 161) = 2.70, *p* = .101. Cramér’s *V* = 0.14 |  |  |
| **F30-F39 diagnoses** | | |  |  |  |  |  |
| Yes | | | 40 (63.5%) | 62 (63.3%) | *χ*²(1, *N* = 161) = 0.00, *p* = 1.000. Cramér’s *V* = 0.00 |  |  |
| **F40-F48 diagnoses** | | |  |  |  |  |  |
|  |  |  |  |  |  |  |  |
| Yes | | | 26 (41.3%) | 42 (42.9%) | *χ*²(1, *N* = 161) = 0.00, *p* = .972. Cramér’s *V* = 0.02 |  |  |
| **F50-F59 diagnoses** | | |  |  |  |  |  |
| Yes | | | 8 (12.7%) | 12 (12.2%) | *χ*²(1, *N* = 161) = 0.00, *p* = 1.000. Cramér’s *V* = 0.01 |  |  |
| **F60-F69 diagnoses** | | |  |  |  |  |  |
| Yes | | | 24 (38.1%) | 40 (40.8%) | *χ*²(1, *N* = 161) = 0.03, *p* = .858. Cramér’s *V* = 0.03 |  |  |
| **F90-F98 diagnoses** | | |  |  |  |  |  |
|  |  |  |  |  |  |  |  |
| Yes | | | 12 (19.0%) | 30 (30.6%) | *χ*²(1, *N* = 161) = 2.09, *p* = .148. Cramér’s *V* = 0.13 |  |  |
| **ACTH** | | |  |  |  |  |  |
| Mean (SD) | | | 17.5 (9.65) | 17.2 (9.78) | *t*(123.12) = 0.18, *p* = .857, *d* = 0.03 |  |  |
|  |  |  |  |  |  |  |  |
| Missing | | | 5 (7.9%) | 9 (9.2%) |  |  |  |
| **TSH** | | |  |  |  |  |  |
| Mean (SD) | | | 2.22 (1.32) | 2.10 (0.980) | *t*(95.98) = 0.60, *p* = .552, *d* = 0.11 |  |  |
|  |  |  |  |  |  |  |  |
| Missing | | | 5 (7.9%) | 5 (5.1%) |  |  |  |
| **fT3** | | |  |  |  |  |  |
| Mean (SD) | | | 3.43 (0.410) | 3.42 (0.365) | *t*(110.49) = 0.15, *p* = .884, *d* = 0.04 |  |  |
| Missing | | | 5 (7.9%) | 5 (5.1%) |  |  |  |
| **DHEA** | | |  |  |  |  |  |
| Mean (SD) | | | 1.89 (1.10) | 1.98 (1.06) | *t*(119.31) = –0.50, *p* = .618, *d* = –0.09 |  |  |
| Missing | | | 5 (7.9%) | 10 (10.2%) |  |  |  |
| **Pain threshold** | | |  |  |  |  |  |
| Mean (SD) | | | 42.7 (3.89) | 42.7 (3.82) | *t*(128.14) = 0.03, *p* = .973, *d* = 0.01. |  |  |
| Missing | | | 1 (1.6%) | 0 (0%) |  |  |  |
| **HRV (rMSSD)** | | |  |  |  |  |  |
| Mean (SD) | | | 49.3 (23.6) | 58.7 (31.1) | *t*(148.79) = –2.13, *p* = .035, *d* = –0.33 |  |  |
|  |  |  |  |  |  |  |  |
| Missing | | | 2 (3.2%) | 5 (5.1%) |  |  |  |

**Note.** BL = baseline; FU = follow-up; NSSI = non-suicidal self-injury; BPD = borderline personality disorder; ACTH = adrenocorticotropic hormone; TSH = thyroid-stimulating hormone; fT3 = free triiodothyronine; DHEA = dehydroepiandrosterone; rMSSD = root mean square of successive differences (resting heart rate variability). Group differences between participants included in the analyses (BL and ≥1 FU) and those with baseline data only were examined using independent samples *t*-tests for continuous variables and χ²-tests for categorical variables. Effect sizes are reported as Cohen’s *d* and Cramér’s V. Missing values are reported as *n* (%).

*Table S3. Variable importance for the model predicting SA using 6 biomarkers.*

| Predictors | ***OR*** | ***95% CI*** | ***LR importance*** | ***EL importance*** | ***SVM importance*** |
| --- | --- | --- | --- | --- | --- |
| **fT3 [ng/l]** | **0.260** | **0.032 – 2.142** | **85/100** | **79/100** | **74/100** |
| **TSH [mUl/l]** | **0.343** | **0.098-1.200** | **15/100** | **21/100** | **12/100** |
| DHEA-S [µg/ml] | 0.605 | 0.259-1.414 | 0/100 | 0/100 | 9/100 |
| Pain  threshold | 1.195 | 0.941 – 1.518 | 0/100 | 0/100 | 4/100 |
| ACTH [pg/ml] | 0.988 | 0.896– 1.089 | 0/500 | 0/500 | 1/100 |
| rHRV | 1.003 | 0.900-1.090 | 0/100 | 0/100 | 0/100 |

**Notes:** SA = Suicide attempts, OR odds ratio, CI confidence interval, fT3 free triiodothyronine, ACTH adrenocorticotropic hormone, DHEA-S dehydroepiandrosterone-sulfate, TSH thyroid stimulating hormone, and rHRV resting heart rate variability. Importance scores represent the number of times each variable was identified as the most important predictor across 100 imputed datasets for each model. For instance, "82/100" in the "LR Importance" column for TSH [mU/l] indicates that TSH was the top-ranked variable in 82 out of 100 imputations for the Logistic Regression model. These scores provide a measure of the consistency with which each variable was ranked as the most important predictor across all imputations for each model (LR Logistic Regression, EL Elastic Net, SVM Support Vector Machine). The best predictors have been marked in bold.

*Table S4. Variable importance for the model predicting SA using 6 biomarkers, and clinical features.*

| Predictors | ***OR*** | ***95% CI*** | ***LR importance*** | ***EL importance*** | ***SVM importance*** |
| --- | --- | --- | --- | --- | --- |
| **Past SB** | **7.279** | **0.795-66.679** | **58/100** | **94/100** | **74/100** |
| fT3 [mUl/l] | 0.158 | 0.007 – 3.328 | 42/100 | 6/100 | 23/100 |
| TSH [ng/l] | 0.345 | 0.057-2.095 | 4/100 | 0/100 | 3/100 |
| DHEA-S [µg/ml] | 0.483 | 0.139-1.681 | 0/100 | 0/100 | 2/100 |
| Alter | 0.658 | 0.321-1.348 | 0/100 | 0/100 | 0/100 |
| Pain threshold | 1.100 | 0.830 – 1.461 | 0/100 | 0/100 | 1/100 |
| BPD symptoms | 1.534 | 0.938 – 2.508 | 0/100 | 0/100 | 0/100 |
| ACTH [pg/ml] | 1.010 | 0.892-1.143 | 0/100 | 0/100 | 0/100 |
| rHRV | 1.014 | 0.966-1.064 | 0/100 | 0/100 | 0/100 |

**Notes:** SA= Suicide attempts, OR odds ratio, CI confidence interval, fT3 free triiodothyronine, ACTH adrenocorticotropic hormone, DHEA-S dehydroepiandrosterone-sulfate, TSH thyroid stimulating hormone, and rHRV resting heart rate variability. Importance scores represent the number of times each variable was identified as the most important predictor across 100 imputed datasets for each model. For instance, "237/500" in the "LR Importance" column for past suicide attempts indicates that TSH was the top-ranked variable in 99 out of 100 imputations for the Logistic Regression model. These scores provide a measure of the consistency with which each variable was ranked as the most important predictor across all imputations for each model (LR Logistic Regression, EL Elastic Net, GB Gradient Boosting, RF Random Forest). The best predictor has been marked in bold.

*Table S5. Models predicting SA using BPD symptoms, and age*

| Models | Metric | Pooled | SD | LB | UB |
| --- | --- | --- | --- | --- | --- |
| Logistic Regression | AUC | 0.786 | 0.138 | 0.737 | 0.829 |
|  | Accuracy | 0.755 | 0.007 | 0.742 | 0.768 |
|  | Brier Score | 0.139 | 0.002 | 0.137 | 0.143 |
|  | Sensitivity | 0.132 | 0.018 | 0.100 | 0.173 |
|  | Specificity | 0.902 | 0.008 | 0.885 | 0.917 |
|  | PPV | 0.241 | 0.026 | 0.193 | 0.296 |
|  | NPV | 0.815 | 0.003 | 0.809 | 0.822 |
| Elastic Net Regression | AUC | 0.787 | 0.165 | 0.727 | 0.836 |
|  | Accuracy | 0.758 | 0.010 | 0.738 | 0.777 |
|  | Brier Score | 0.139 | 0.002 | 0.136 | 0.142 |
|  | Sensitivity | 0.123 | 0.016 | 0.095 | 0.159 |
|  | Specificity | 0.908 | 0.014 | 0.877 | 0.932 |
|  | PPV | 0.239 | 0.027 | 0.189 | 0.298 |
|  | NPV | 0.815 | 0.003 | 0.809 | 0.820 |
| Support Vector Machine | AUC | 0.588 | 0.296 | 0.442 | 0.720 |
|  | Accuracy | 0.796 | 0.009 | 0.777 | 0.813 |
|  | Brier Score | 0.155 | 0.002 | 0.153 | 0.159 |
|  | Sensitivity | 0.003 | 0.009 | 0.000 | 0.813 |
|  | Specificity | 0.983 | 0.016 | 0.896 | 0.998 |
|  | PPV | 0.020 | 0.097 | 0.000 | 0.998 |
|  | NPV | 0.808 | 0.001 | 0.805 | 0.811 |

**Notes:** SA = Suicide attempts; BPD = Borderline personality disorder, SD = Standard deviation. Reported performance metrics include AUC (area under the receiver operating characteristic curve), Accuracy, F1 Score (harmonic mean of precision and recall), Brier Score (mean squared difference between predicted probabilities and actual outcomes), Sensitivity (true positive rate), Specificity (true negative rate), PPV (positive predictive value), and NPV (negative predictive value). A model with AUC = 1 is considered perfect, 0.9 < AUC ≤ 1 as excellent, 0.8 < AUC ≤ 0.9 as good, 0.7 < AUC ≤ 0.8 as fair, 0.6 < AUC ≤ 0.7 as poor, and 0.5 < AUC ≤ 0.6 as slightly better than chance. LB and UB refer to the lower and upper bounds of the 95% confidence intervals (CIs), respectively.

*Table S6. Models predicting SA using, BPD symptoms, past suicide attempts, age.*

| Models | Metric | Pooled | SD | LB | UB |
| --- | --- | --- | --- | --- | --- |
| Logistic Regression | AUC | 0.788 | 0.165 | 0.729 | 0.838 |
|  | Accuracy | 0.815 | 0.007 | 0.801 | 0.828 |
|  | Brier Score | 0.128 | 0.002 | 0.125 | 0.131 |
|  | Sensitivity | 0.380 | 0.022 | 0.338 | 0.425 |
|  | Specificity | 0.917 | 0.007 | 0.903 | 0.929 |
|  | PPV | 0.519 | 0.024 | 0.472 | 0.566 |
|  | NPV | 0.863 | 0.004 | 0.854 | 0.871 |
| Elastic Net Regression | AUC | 0.799 | 0.171 | 0.738 | 0.848 |
|  | Accuracy | 0.810 | 0.006 | 0.798 | 0.821 |
|  | Brier Score | 0.128 | 0.002 | 0.123 | 0.133 |
|  | Sensitivity | 0.077 | 0.165 | 0.001 | 0.894 |
|  | Specificity | 0.983 | 0.035 | 0.468 | 1.000 |
|  | PPV | 0.504 | 0.644 | 0.006 | 0.994 |
|  | NPV | 0.827 | 0.022 | 0.779 | 0.867 |
| Support Vector Machine | AUC | 0.703 | 0.296 | 0.569 | 0.810 |
|  | Accuracy | 0.797 | 0.006 | 0.784 | 0.809 |
|  | Brier Score | 0.151 | 0.002 | 0.148 | 0.155 |
|  | Sensitivity | 0.239 | 0.058 | 0.142 | 0.372 |
|  | Specificity | 0.928 | 0.013 | 0.898 | 0.949 |
|  | PPV | 0.437 | 0.037 | 0.365 | 0.511 |
|  | NPV | 0.839 | 0.008 | 0.823 | 0.853 |

**Note.** SA = Suicide attempts; BPD = Borderline personality disorder, SD = Standard deviation. Reported performance metrics include AUC (area under the receiver operating characteristic curve), Accuracy, F1 Score (harmonic mean of precision and recall), Brier Score (mean squared difference between predicted probabilities and actual outcomes), Sensitivity (true positive rate), Specificity (true negative rate), PPV (positive predictive value), and NPV (negative predictive value). A model with AUC = 1 is considered perfect, 0.9 < AUC ≤ 1 as excellent, 0.8 < AUC ≤ 0.9 as good, 0.7 < AUC ≤ 0.8 as fair, 0.6 < AUC ≤ 0.7 as poor, and 0.5 < AUC ≤ 0.6 as slightly better than chance. LB and UB refer to the lower and upper bounds of the 95% confidence intervals (CIs), respectively.

*Table S7. Models predicting NSSI remissions using BPD symptoms, past NSSI, and age.*

| Models | Metric | Pooled | SD | LB | UB |
| --- | --- | --- | --- | --- | --- |
| Logistic Regression | AUC | 0.404 | 0.138 | 0.340 | 0.471 |
|  | Accuracy | 0.418 | 0.015 | 0.388 | 0.449 |
|  | Brier Score | 0.274 | 0.003 | 0.269 | 0.278 |
|  | Sensitivity | 0.434 | 0.024 | 0.388 | 0.482 |
|  | Specificity | 0.402 | 0.019 | 0.364 | 0.441 |
|  | PPV | 0.413 | 0.016 | 0.382 | 0.444 |
|  | NPV | 0.423 | 0.016 | 0.392 | 0.454 |
| Elastic Net Regression | AUC | 0.500 | 0.140 | 0.431 | 0.569 |
|  | Accuracy | 0.484 | 0.003 | 0.479 | 0.489 |
|  | Brier Score | 0.250 | 0.000 | 0.250 | 0.250 |
|  | Sensitivity | 0.291 | 0.032 | 0.232 | 0.358 |
|  | Specificity | 0.671 | 0.036 | 0.597 | 0.738 |
|  | PPV | 0.462 | 0.000 | 0.462 | 0.462 |
|  | NPV | 0.494 | 0.002 | 0.490 | 0.498 |
| Support Vector Machine | AUC | 0.593 | 0.165 | 0.513 | 0.669 |
|  | Accuracy | 0.456 | 0.019 | 0.419 | 0.493 |
|  | Brier Score | 0.248 | 0.001 | 0.245 | 0.251 |
|  | Sensitivity | 0.270 | 0.087 | 0.133 | 0.472 |
|  | Specificity | 0.635 | 0.114 | 0.395 | 0.822 |
|  | PPV | 0.419 | 0.021 | 0.378 | 0.460 |
|  | NPV | 0.472 | 0.019 | 0.435 | 0.509 |

**Note.** BPD = Borderline personality disorder. NSSI = non-suicidal self-injury. Reported performance metrics include AUC (area under the receiver operating characteristic curve), Accuracy, F1 Score (harmonic mean of precision and recall), Brier Score (mean squared difference between predicted probabilities and actual outcomes), Sensitivity (true positive rate), Specificity (true negative rate), PPV (positive predictive value), and NPV (negative predictive value). A model with AUC = 1 is considered perfect, 0.9 < AUC ≤ 1 as excellent, 0.8 < AUC ≤ 0.9 as good, 0.7 < AUC ≤ 0.8 as fair, 0.6 < AUC ≤ 0.7 as poor, and 0.5 < AUC ≤ 0.6 as slightly better than chance. LB and UB refer to the lower and upper bounds of the 95% confidence intervals (CIs), respectively.

**2. Figures**

Figure S1. AUC for models predicting suicidal behavior using BPD symptoms and age.

Notes: The bars represent the performance of machine learning models: A model with AUC = 1 would be considered as perfect, 0.9 < AUC < 1 as excellent, 0.8 < AUC < 0.9 as good, 07 < AUC < 0.8 as fair, 0.6 < AUC < 0.7 as poor and 0.5 < AUC < 0.6 as being slightly better than chance. The error bars represent the 95% confidence intervals (CIs) of AUC values.

Figure S2. AUC for models predicting suicidal behavior using BPD symptoms, past suicidal behavior, and age.

Notes: The bars represent the performance of machine learning models: A model with AUC = 1 would be considered as perfect, 0.9 < AUC < 1 as excellent, 0.8 < AUC < 0.9 as good, 07 < AUC < 0.8 as fair, 0.6 < AUC < 0.7 as poor and 0.5 < AUC < 0.6 as being slightly better than chance. The error bars represent the 95% confidence intervals (CIs) of AUC values.

Figure S3. NSSI = non-suicidal self-injury, AUC for models predicting NSSI remissions using BPD, past NSSI, and age in females.

Notes: The bars represent the performance of machine learning models: A model with AUC = 1 would be considered as perfect, 0.9 < AUC < 1 as excellent, 0.8 < AUC < 0.9 as good, 07 < AUC < 0.8 as fair, 0.6 < AUC < 0.7 as poor and 0.5 < AUC < 0.6 as being slightly better than chance. The error bars represent the 95% confidence intervals (CIs) of AUC values.
